# Excitation of “forbidden” guided-wave plasmon polariton modes via direct reflectance using a low refractive index polymer coupling layer

**DOI:** 10.1371/journal.pone.0276522

**Published:** 2022-10-26

**Authors:** Colin D. Marquis, Lindze M. McCarley, Amy L. Pollock, Acamaro S. Cutcher, Max T. Cannella, Tierra L. Smith, Michael B. Larsen, Brandon M. Peden, Brad L. Johnson, Janelle M. Leger

**Affiliations:** 1 Department of Physics and Astronomy, Western Washington University, Bellingham, Washington, United States of America; 2 Department of Chemistry, Western Washington University, Bellingham, Washington, United States of America; Universiti Brunei Darussalam, BRUNEI DARUSSALAM

## Abstract

A surface plasmon polariton (SPP) is an excitation resulting from the coupling of light to a surface charge oscillation at a metal-dielectric interface. The excitation and detection of SPPs is foundational to the operating mechanism of a number of important technologies, most of which require SPP excitation via direct reflectance, commonly achieved via Attenuated Total Reflection (ATR) using the Kretschmann configuration. As a result, the accessible modes are fundamentally high-loss “leaky modes,” presenting a critical performance barrier. Recently, our group provided the first demonstration of “forbidden,” or guided-wave plasmon polariton modes (GW-PPMs), collective modes of a MIM structure with oscillatory electric field amplitude in the central insulator layer with up to an order of magnitude larger propagation lengths than those of traditional SPPs. However, in that work, GW-PPMs were accessed by indirect reflectance using Otto configuration ATR, making them of limited applied relevance. In this paper, we demonstrate a technique for direct reflectance excitation and detection of GW-PPMs. Specifically, we replace the air gap used in traditional Otto ATR with a low refractive index polymer coupling layer, mirroring a technique previously demonstrated to access Long-Range Surface Plasmon Polariton modes. We fit experimental ATR data using a robust theoretical model to confirm the character of the modes, as well as to explore the potential of this approach to enable advantageous propagation lengths. The ability to excite GW-PPMs using a device configuration that does not require an air gap could potentially enable transformative performance enhancements in a number of critical technologies.

## Introduction

One method that has been demonstrated to circumvent the diffraction limit in optical signal processing is to couple a photon to a surface charge-oscillation excitation at a metal-dielectric interface to create a coupled excitation known as a surface plasmon polariton (SPP) [1–11]. Technologies that utilize SPPs are maturing rapidly and include sensors and optical devices for information technology, among others [12–14]. However, the development of these technologies has been limited by the substantial propagation losses associated with SPPs. Specifically, the energy of a SPP is typically concentrated within approximately one skin depth of the metal-dielectric interface, resulting in large Ohmic losses within the metal layer. Progress has been made in increasing the propagation length of SPP modes through strategic structural designs that move more of the electric field away from the metal film [15–30]. However, this comes at the cost of structural complexity, and the useful range of a typical plasmonic excitation remains in the micrometer range.

**Fig 1:**
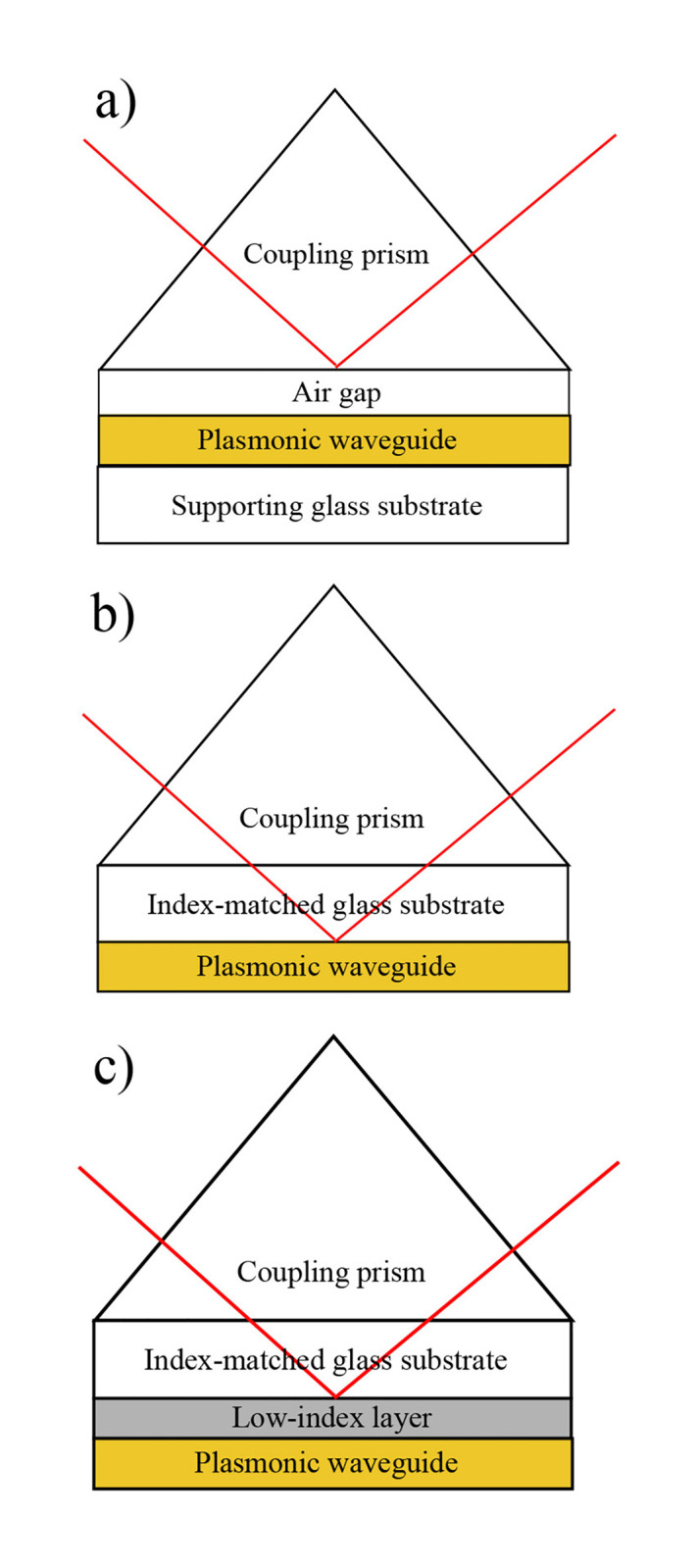
Schematic representations of attenuated total reflection. a) the Otto configuration; b) the Kretschmann configuration; c) the Kretschmann configuration with the addition of a low-index coupling layer, as reported here.

An important consideration in the design of structures supporting SPP modes is the mechanism of excitation. Specifically, SPPs are excited and detected using Attenuated Total Reflection (ATR) in one of two experimental configurations, known as the Otto and Kretschmann configurations (Fig 1a and 1b, respectively) [31, 32]. Importantly, these configurations access different regions of dispersion space for which the plasmonic modes have distinctly different characters. Most fundamental studies of SPPs utilize the Otto configuration, an indirect reflectance technique, wherein the plasmon-supporting structure is separated from a coupling prism using an air gap. However, most plasmonic applications require device structures for which mode excitation occurs via direct reflectance, necessitating the use of the Kretschmann ATR configuration, in which the plasmonic element is deposited either directly on a coupling prism, or on a substrate that is index-matched to a coupling prism. Practically speaking, this means that the plasmonic modes accessible to these applications are limited to the region of dispersion space that supports “leaky modes.” Specifically, the amplitude of the electric field profile as a function of position along the propagating interface is proportional to $e^{ik_{x}x}$, where *x* is the direction of propagation. While non-leaky modes generally have a purely real *k*_*x*_ and therefore theoretically limitless propagation, for leaky modes *k*_*x*_ has a large imaginary component, leading to fundamentally high losses, even in the case of an ideal (lossless) supporting structure [7, 33–36]. This practical limitation further exacerbates the problem of high propagation losses in SPPs.

**Fig 2:**
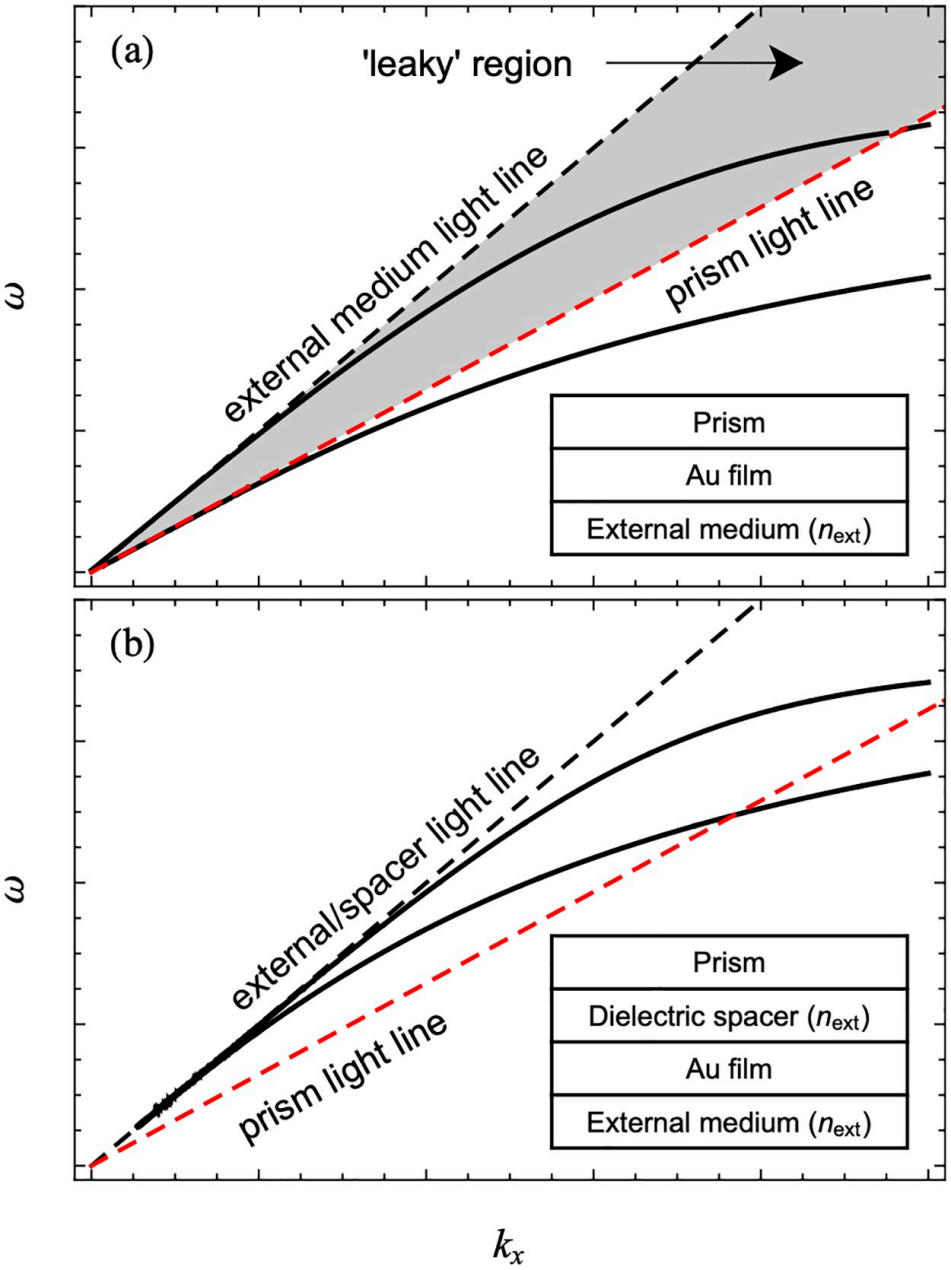
Dispersion relationships for a single gold film. a) the standard Kretschmann configuration; b) the Kretschmann configuration with the addition of a dielectric spacer layer. Shown are the SPP modes (black) and the relevant light lines. The area shaded grey is the region in which leaky modes are found.

One approach that has been demonstrated to improve propagation lengths in an applications-relevant geometry is the use of structures that support Long-Range Surface Plasmon Polariton (LRSPP) modes [19, 37–43]. In contrast to traditional SPPs accessed using the Kretschmann configuration, LRSPPs are accessed by inserting a dielectric spacer layer with a refractive index as close as possible to that of the external dielectric layer (on the side of the waveguide opposite that of the prism, typically air or water) between the prism and the metal film (Fig 1c). This subtle difference significantly impacts the properties of the plasmonic modes supported by the structure. Briefly, the standard Kretschmann ATR is restricted to the detection of leaky modes because the substrate and prism are the same in this configuration. Specifically, the accessible modes (those lying to the left of the prism light line in the dispersion plot) necessarily lie to the left of the prism-side dielectric light line as well. This relationship is what leads to complex values for *k*_*x*_ as indicated above (Fig 2a). In contrast, when a dielectric film with a refractive index matching that of the external dielectric layer (and therefore a lower refractive index than the prism) is inserted between the substrate and the plasmonic film, a region of dispersion space becomes accessible in which the modes lie to the right of the external dielectric/spacer layer light line (Fig 2b). As a consequence, the modes accessed in this configuration have a real *k*_*x*_ in the absence of damping and are therefore non-leaky plasmon modes that are capable of demonstrating significantly increased propagation lengths in comparison with leaky modes. In this case, the two plasmon modes localized at the two metal interfaces form a symmetric (lower branch) and an antisymmetric (upper branch) combination in the coupled system. The lower branch generally has a higher propagation length and is referred to as the LRSPP. The fundamental approach of influencing the properties of accessible plasmonic modes by manipulating the dispersion space available for a particular device structure will be relevant to the work presented here.

In an effort unrelated to the development of LRSPPs, our group recently provided the first experimental demonstration of so-called “forbidden” or guided-wave plasmon polariton modes (GW-PPMs), also referred to as high-index dielectric PPMs [44, 45]. GW-PPMs are supported by a metal-insulator-metal (MIM) structure (having dimensions smaller than the excitation wavelength) with a high refractive index insulator layer (in this case TiO_2_) in place of the standard SiO_2_. Because the central insulator layer has a higher refractive index than the supporting substrate, it creates a distinct region of phase space in the dispersion diagram between the TiO_2_ light line and substrate light line in which GW-PPMs, collective plasmon-polariton modes with oscillatory electric field amplitude in the central insulator layer, can be found [44]. A key feature of these modes is that the wavelength of the oscillatory field within the TiO_2_ is independent of the excitation wavelength. Importantly, we found that GW-PPM propagation lengths are sensitive to both the insulator layer thickness and the parallel component of the wavevector, allowing for modes within the GW-PPM phase space with up to an order of magnitude larger propagation lengths than those of traditional SPPs. However, similar to LRSPPs, GW-PPMs are accessed in a region of phase space not accessible by direct reflectance using the standard Kretschmann configuration ATR, and are therefore of limited practical relevance as previously reported.

In this article we present the use of a low-index dielectric polymer coupling layer for the excitation and detection of GW-PPMs using the Kretschmann ATR configuration. We use theoretical fittings of ATR spectra to produce a dispersion plot relevant to our system, and in turn use the dispersion calculation to generate electric field profiles and propagation lengths. We confirm that this approach yields modes with guided-wave character, and that these GW-PPMs have significant potential advantages in terms of propagation length.

## Methods

**Fig 3:**
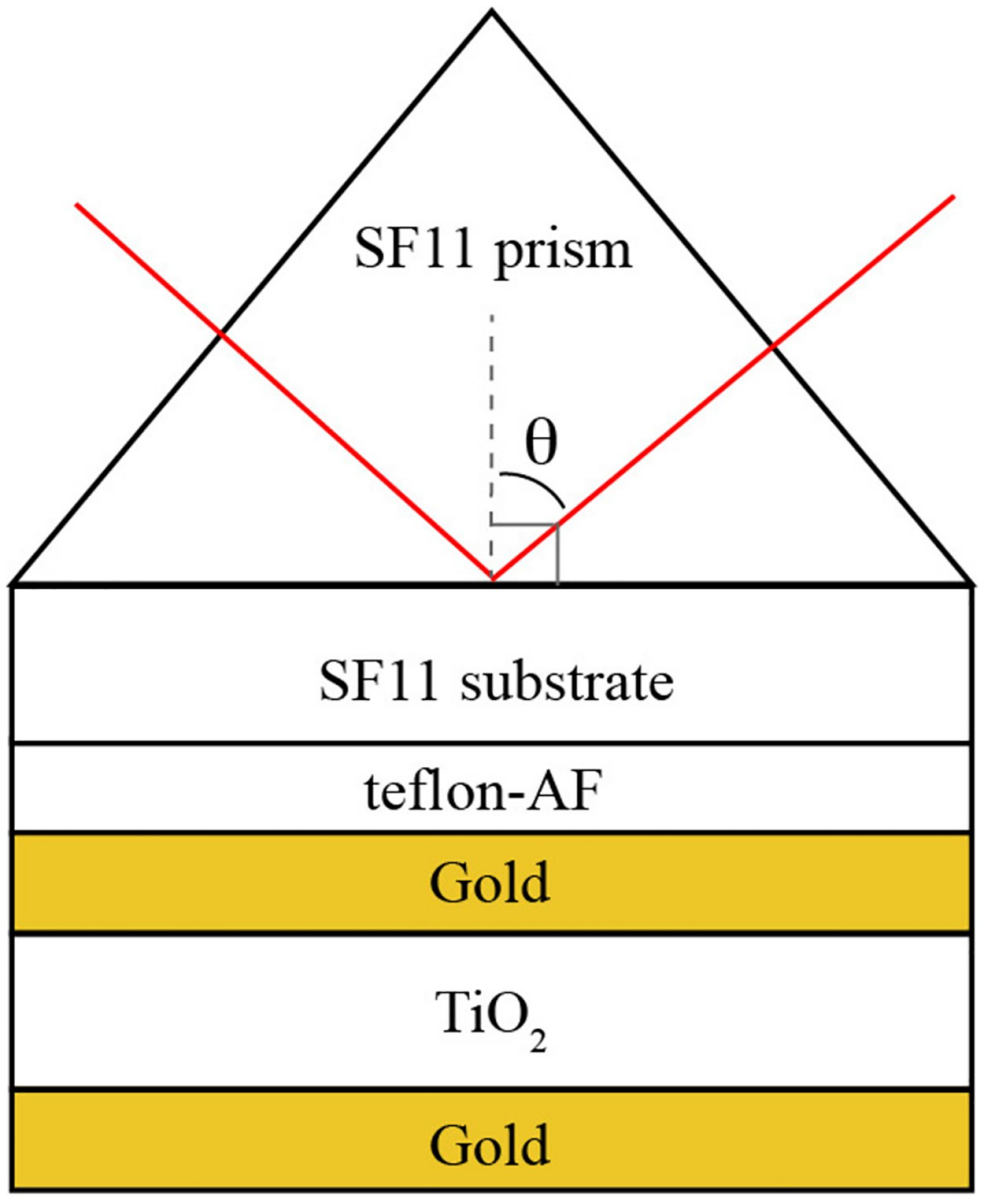
Schematic structure of the device reported in this study showing the defined internal angle.

Samples were constructed using the structure shown in Fig 3. SF11 substrates (Newlight Photonics, *n* = 1.78) were first cleaned by sonication, stepping through a sequential wash cycle of dilute glass detergent, DI water, acetone, and isopropanol at 30°C for 15 minutes at each sonication step. After sonication, substrates were dried using pressurized air, then transferred to a PDC-32G plasma cleaner for a 5-minute argon plasma cleaning. Clean substrates were kept under inert atmosphere during storage. The low refractive index coupling layer was created by spin-coating a solution of Teflon AF 1600 (Sigma-Aldrich) which was dissolved at 2% by weight in perfluoro(2-butyl tetrahydrofuran). Prior to spin-coating, full dissolution typically required a minimum of 24 h. For the sample data reported here, a Teflon-AF layer with a thickness of 220 nm was used. The refractive index of the resulting polymer film was determined using a Jasco V-670 UV-Vis-NIR spectrometer equipped with a Jasco ISN-723 integrating sphere. Briefly, reflectance spectra of polymer films were transformed with Jasco Spectral Analysis software using Kramers-Kronig relations, with the transparent region set to 500–800 nm. The resulting refractive index was consistent with literature (n = 1.3) [46]. Film thicknesses were measured using a Bruker Bioscope Catalyst atomic force microscope (AFM) in tapping mode. Gold films were deposited using thermal evaporation performed at a pressure of 8x10^-6^ mbar. TiO_2_ layers were deposited using AC sputter coating using a 99.9% pure TiO_2_ sputter target from Kurt J. Lesker Company, Ltd. at 1x10^-2^ mbar under argon atmosphere. For each of the depositions a blank substrate was included to separately verify thickness of the layers via AFM. Surface roughness for the various layers typically fall in the range of 1–3 nm.

**Fig 4:**
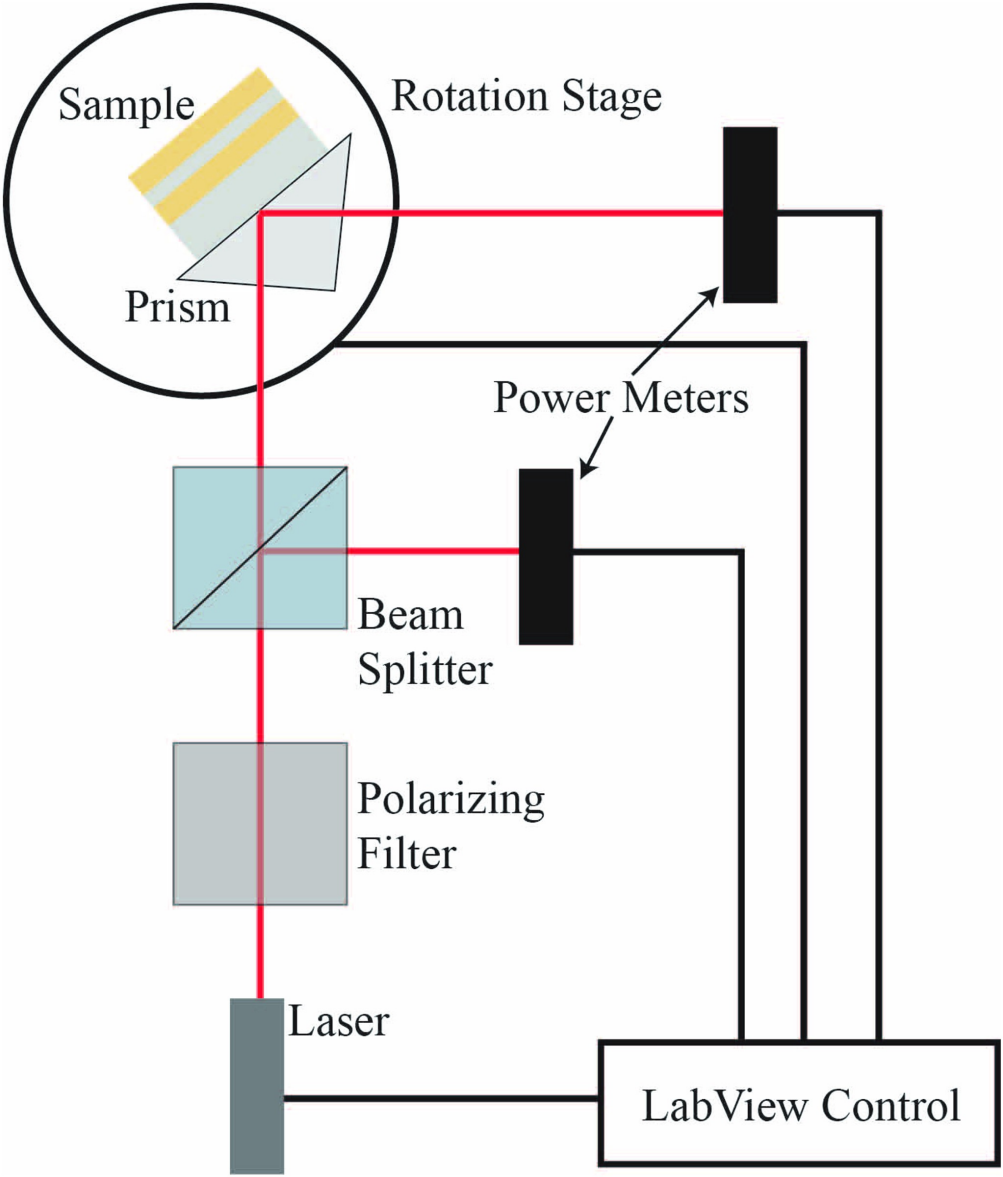
Schematic of the attenuated total reflectance experimental setup used in this study.

Excitation and detection of the plasmonic modes were carried out using ATR with an Edmunds Optics SF11 right-angle prism and a Toptica iCrome TVIS laser, tunable for 488–640 nm. The incident beam was p-polarized using a Glan-Taylor calcite polarizing prism (ThorLabs). The beam was then split, the power of the first beam was measured directly, while the other was measured following reflection off the prism. The ratio of the power between the two beams was calibrated before testing. The internal angle of the beam incident to the plane of the waveguide was controlled using a servo-controlled rotation stage. Using a Thorlab stepped motor rotation stage, reflectance data were collected as a function of excitation wavelength for various internal angles (from 61° to 50°) in one-degree intervals (note that the critical angle in this system is approximately 47°). Dispersion in the refractive index of the prism was accounted for in real time by adjusting the external angle in order to keep the internal angle consistent during a scan. A single drop of diiodomethane (Acros Organics) was used as an index matching fluid (*n* = 1.74) between the SF11 prism and substrate. Slight pressure applied to the sample resulted in an even spread of the index matching fluid and aided adhesion to the surface of the prism. Substrates were situated in contact in the modified Kretschmann configuration as shown in Fig 4.

We computed theoretical ATR scans and extracted structural device parameters (including layer thicknesses and others) by fitting the simulated ATR to the experimental scans. These parameters were used to compute the dispersion relations describing the resonant modes of the Teflon/Au/TiO_2_/Au/air structure. By correlating the position of the minima of the ATR scans to points in the dispersion relation in *ω*-*k* space, we extracted the propagation lengths associated with the excited mode. The propagation lengths were computed as 2/Im(*k*_*x*_), where *k*_*x*_ is the complex wave vector of the mode whose frequency matches that of the minimum of the ATR scan. To compute both the ATR and dispersion relations, we solved the electromagnetic wave equation in each layer by assuming that the solution can be written as a sum of counter-propagating plane waves perpendicular to the interfaces, subject to the condition that the displacement field in each region be divergenceless, as described previously [45]. We then matched the solutions in consecutive layers by ensuring that the electromagnetic boundary conditions are satisfied across each interface. These conditions can be combined into a homogeneous matrix equation for the field amplitudes for computing dispersion relations, or an inhomogeneous matrix equation with an incoming wave in the prism as a source for the case of computing ATR and solved numerically. The dielectric response of each layer can be modeled via a dielectric function. We treated the dielectric layers as having constant, real dielectric functions, whereas we use a frequency-dependent dielectric function for the gold which includes the effects of two optical transitions [47, 48]. Careful details of the calculations and of the dielectric function used to model the gold can be found in Ref. [45].

## Results and discussion

**Fig 5:**
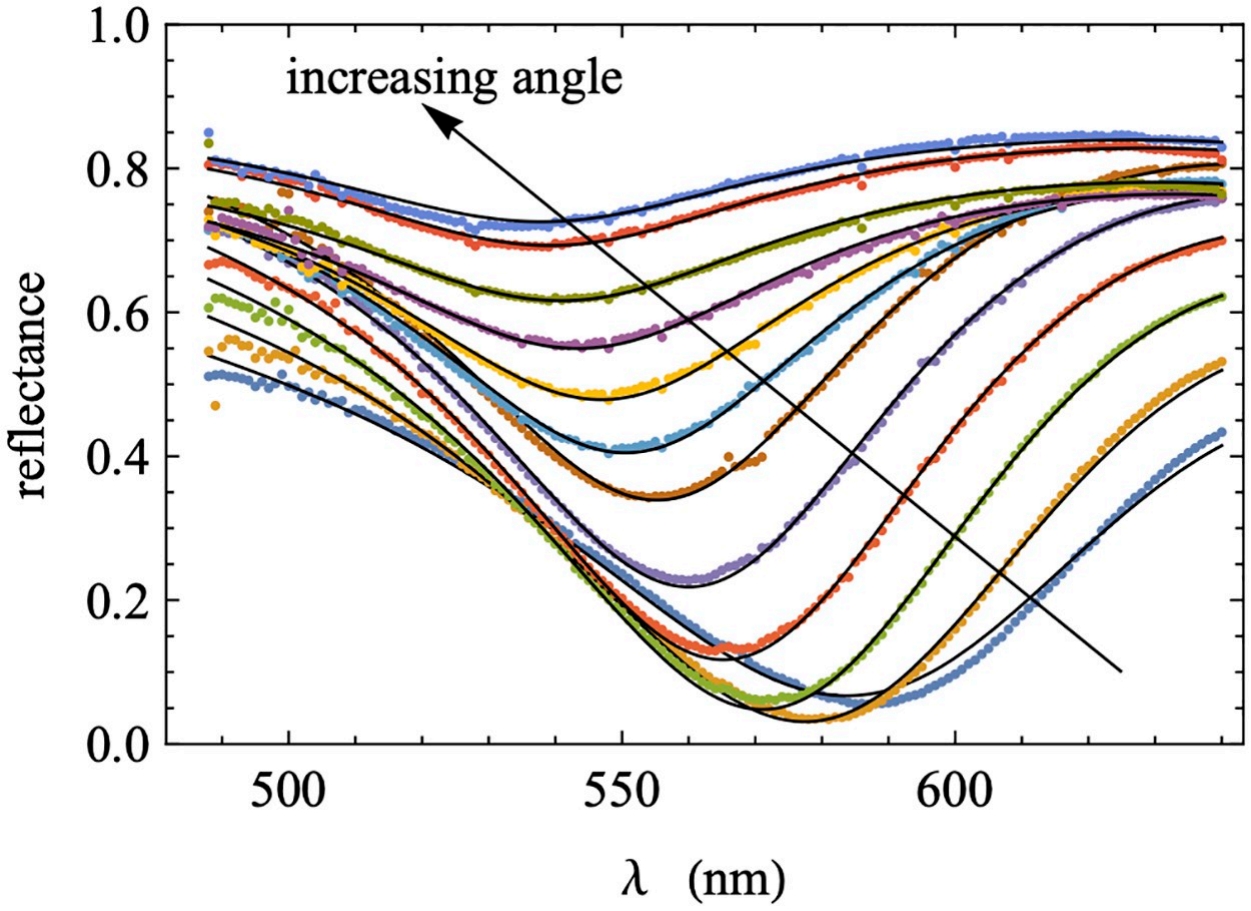
ATR reflectance as a function of excitation wavelength shown for a range of internal angles (50 to 61 degrees in one-degree increments). Shown are experimental measurements (symbols) together with model results (lines) for corresponding internal angles.

The first step in determining the character and properties of the plasmonic modes supported by a specific structure is to perform ATR measurements. The model fits of the reflectance data, when collected over a region of phase space for which minima are observed, provide the physical parameters required to generate a dispersion diagram appropriate for that structure, which will in turn be used to calculate electric field profiles and propagation lengths. The structure depicted in Fig 3 was constructed using layer thicknesses of ***d***_**teflon**_ = **200 nm**, ***d***_**Au,1**_ = **35 nm**, $d_{TiO_{2}}=100\;nm$, and ***d***_**Au,2**_ = **36 nm** as determined via AFM. The target film thicknesses used in this study were chosen with the goal of creating a structure for which the supported plasmonic modes could be excited and detected in a wavelength and angle regime detectable using our specific ATR experimental setup. Fig 5 shows the ATR spectra for this structure as a function of the wavelength of the excitation laser, for internal angles ranging from 50 to 61 degrees. The ATR minima are clearly observed and are seen to become shallower and move to higher frequencies as the internal angle of the laser in the prism is increased. Shown also are the curves obtained by fitting the simulated ATR scans to the experimental scans. Fitting parameters included the angles and thickness of the TiO_2_ layer (due to uncertainties in their measured values), the gold plasma frequency ***ω***_***p***_ and plasma damping ***γ***_***p***_, the dielectric constant ***ϵ***_**T**_ for Teflon-AF, and a uniform reflectance shift for each scan (to account for experimental variation which we attribute to scattering). The values found from the fitting are summarized in Table 1. The TiO_2_ layer thickness matched the value above to within experimental error, and the Drude parameter ***ω***_***p***_ and the dielectric constant ***ϵ***_**T**_ were close to the literature values (8.67 eV and 1.69 respectively) [46, 47]. While not reported in the table, the angles were all within 0.3 degrees of the experimental values. The result for the Drude damping ***γ***_***p***_ is significantly different than the literature value of ***γ***_***p***_ = **0.085** eV by about a factor of two [46, 47]. However, available literature values were measured using bulk samples of gold, while here the gold layers are thin films. We anticipate that surface roughness could possibly have an outsized effect and therefore explain this discrepancy. We note that in our previous work [45], in order to get good fits between experimental and theoretical ATR scans, the plasma damping was similarly always at least 50% larger than the literature value. For more discussion justifying our choice of fitting parameters, see the Supplementary Material.

**Table 1:** Fitting parameters and the values obtained from fitting the ATR scans shown in Fig 5.

Parameter	Fit Value
Teflon thickness	200 nm
1^st^ gold layer thickness	35 nm
TiO_2_ thickness	105 nm
2^nd^ gold layer thickness	36 nm
**ℏ*ω*_*p*_**	8.65 eV
**ℏ*γ*_*p*_**	0.179 eV
***ϵ*_*T*_**	1.64

**Fig 6:**
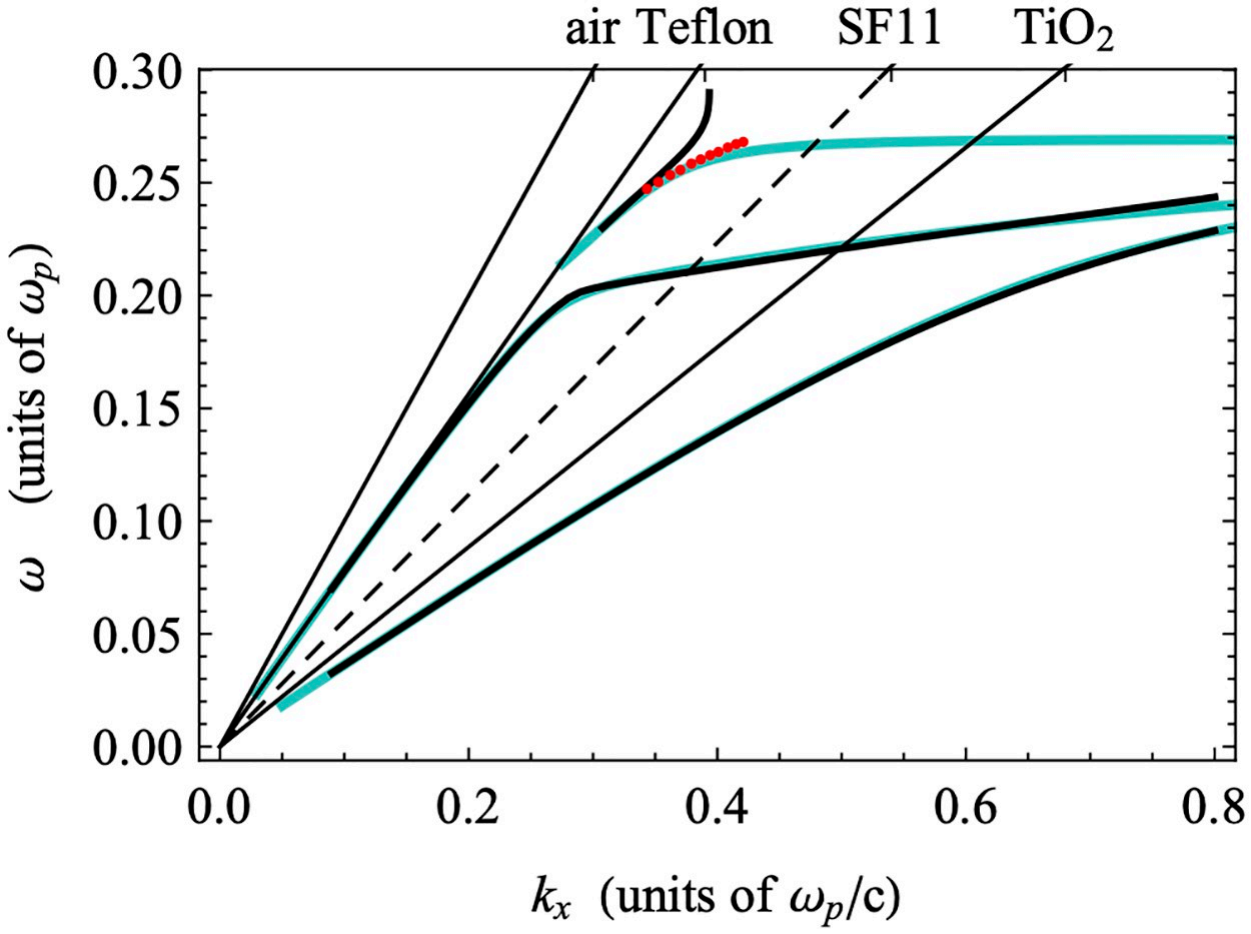
Calculated dispersions generated both with (black lines) and without (blue lines) damping included in the gold dielectric function. Shown are the light lines for the relevant dielectric materials and the minima (red dots) of the measured ATR curves (see Fig 5).

Fig 6 shows the model dispersion computed using the physical parameters generated by the ATR fittings presented in Fig 5. The dispersion calculated with the full gold dielectric function (including both visible transition resonances), both with and without damping, are shown. In addition, the minima of the associated ATR scans are plotted as red points for reference. The undamped dispersion relations (blue) are consistent with previous work on systems with MIM structures [45]. The modes to the right of the TiO_2_ light line are all standard SPP modes. In particular, the lowest frequency mode is a SPP localized to the TiO_2_-Au interface, and the highest-frequency mode in this region is an SPP localized to the Teflon-Au interface. In this work, the region between the Teflon and TiO_2_ light lines is the region of interest, as this is where we expect GW-PPM modes to be supported, as will be described in more detail below.

As previously identified, in the presence of damping in the metal layer, branches of the dispersion relation can display a behavior in which the dispersion “bends back” towards the light line rather than reaching a horizontal SPP resonance asymptote at large ***k***_***x***_ [6, 49, 50]. Specifically, the minima of ATR scans at fixed wavelength tend to display bend-back, and therefore lie along or close to the damped dispersions, while the minima of ATR scans at fixed internal angle tend to lie along or close to the undamped dispersions [49, 50]. As expected for this study, near the region of phase space accessible using our experimental ATR setup, the dispersion relation in the presence of damping (black) displays strong bend-back behavior as compared to the dispersion in the absence of damping (blue), while the minima (red) of the fixed-angle experimental wavelength scans shown in Fig 5 follow the undamped dispersion relation. We are therefore able to associate the measured ATR dip with the higher-energy mode identified in the region of interest.

**Fig 7:**
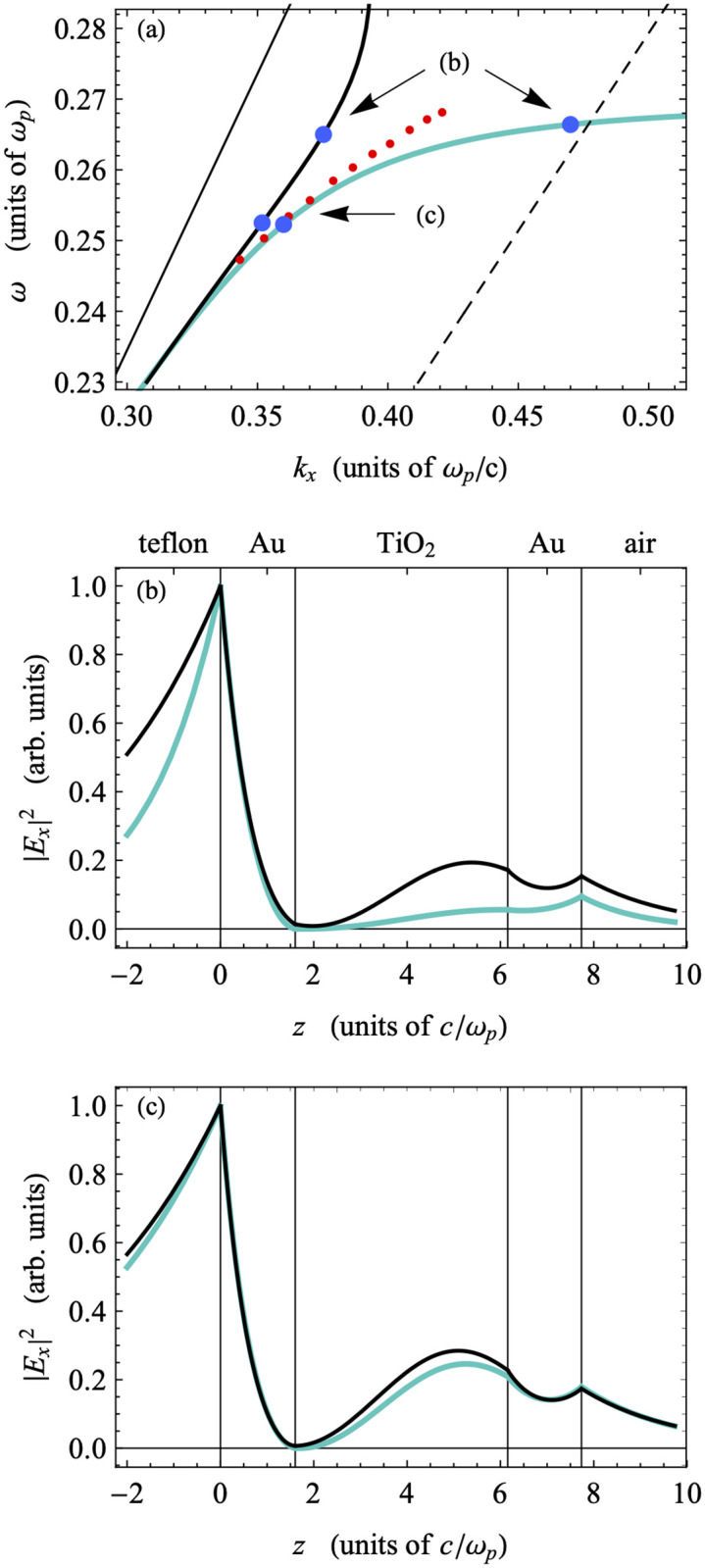
Magnification of experimentally-relevant dispersion region in Fig 6 and electric field amplitudes. a) the experimentally relevant region of the dispersion displayed in Fig 6; b) electric field amplitude (modulus squared) associated with point b on the dispersion plot; c) electric field amplitude (modulus squared) associated with point c on the dispersion plot. Red dots indicate the minima of the experimental ATR scans (see Fig 5).

To confirm the guided-wave character of the mode identified in the ATR measurement, we examine the structure of the fields in the frequency range probed in our experiments. The field amplitudes of the modes shown in the region of dispersion space between the Teflon-AF and TiO_2_ light lines should display the sinusoidal rather than exponential behavior inside the TiO_2_ layer that is characteristic of GW-PPMs [44]. Fig 7a shows a portion of the dispersion from Fig 6, focused in on the experimentally relevant region. Several points are marked on the dispersions that indicate points for which the electric field amplitudes are calculated (Fig 7b and 7c). The two sets of points selected are taken at the same frequencies for each of the damped and undamped dispersions. The fields display the characteristic sinusoidal behavior in the TiO_2_ layer, consistent with GW-PPMs as demonstrated previously [45], and are otherwise localized to the Teflon-Au interface. The structures of the undamped and damped fields are identical, but the fields are more highly localized within the TiO_2_ layer at lower frequencies when the dispersions are nearer to the Teflon light line. The undamped and damped modes are nearly identical at lower frequencies where the minima of the experimental ATR scans match both dispersions. The close match between the fields further justifies the identification of the minimum of an ATR scan with the mode in the damped dispersion with the same frequency as the minimum, for which there is literature precedent (see below).

At lower frequencies, both the damped and undamped dispersions are almost indistinguishable, and the ATR minima clearly track both. Therefore, matching the dip in the ATR scan to a mode is relatively straightforward. However, at higher frequencies, the minima more closely match the undamped dispersion, which significantly deviates from the damped one. Therefore, there is an ambiguity in how we identify the mode corresponding to the dip in the ATR scan. The standard approach to calculating the propagation lengths in these cases involves matching the frequency of the experimental minimum to the frequency of the mode in the dispersion relation calculated including the full dielectric functions that include damping, and then extracting the imaginary part of ***k***_***x***_. However, recent research has called into question the simple identification of resonance features in ATR scans with the normal modes of the isolated structure [34, 51–54]. A more careful analysis in which we monitor the root-pole structure of the complex reflectance ***r***(***k***_***x***_) = ***r***(**Re**(***k***_***x***_) + ***i* Im**(***k***_***x***_)) of the entire physical device (including both damping and the coupling prism) could allow us to identify the modes excited by the in-coupled laser light with less ambiguity. This analysis is the subject of future work.

**Fig 8:**
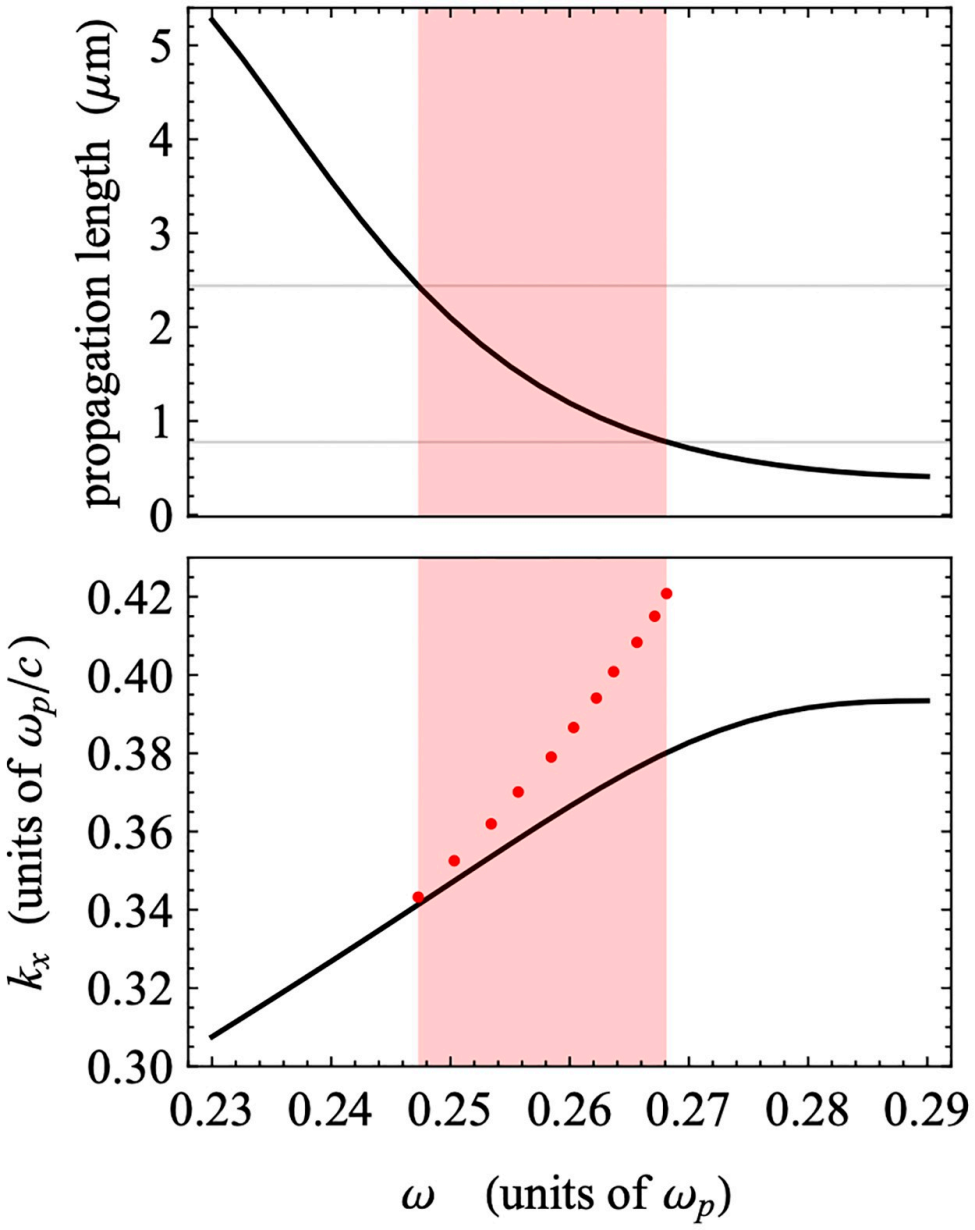
Calculated propagation length as a function of frequency (top panel) and undamped dispersion relationship (bottom panel). Red dots indicate the minima of the experimental ATR scans (see Fig 5).

**Fig 9:**
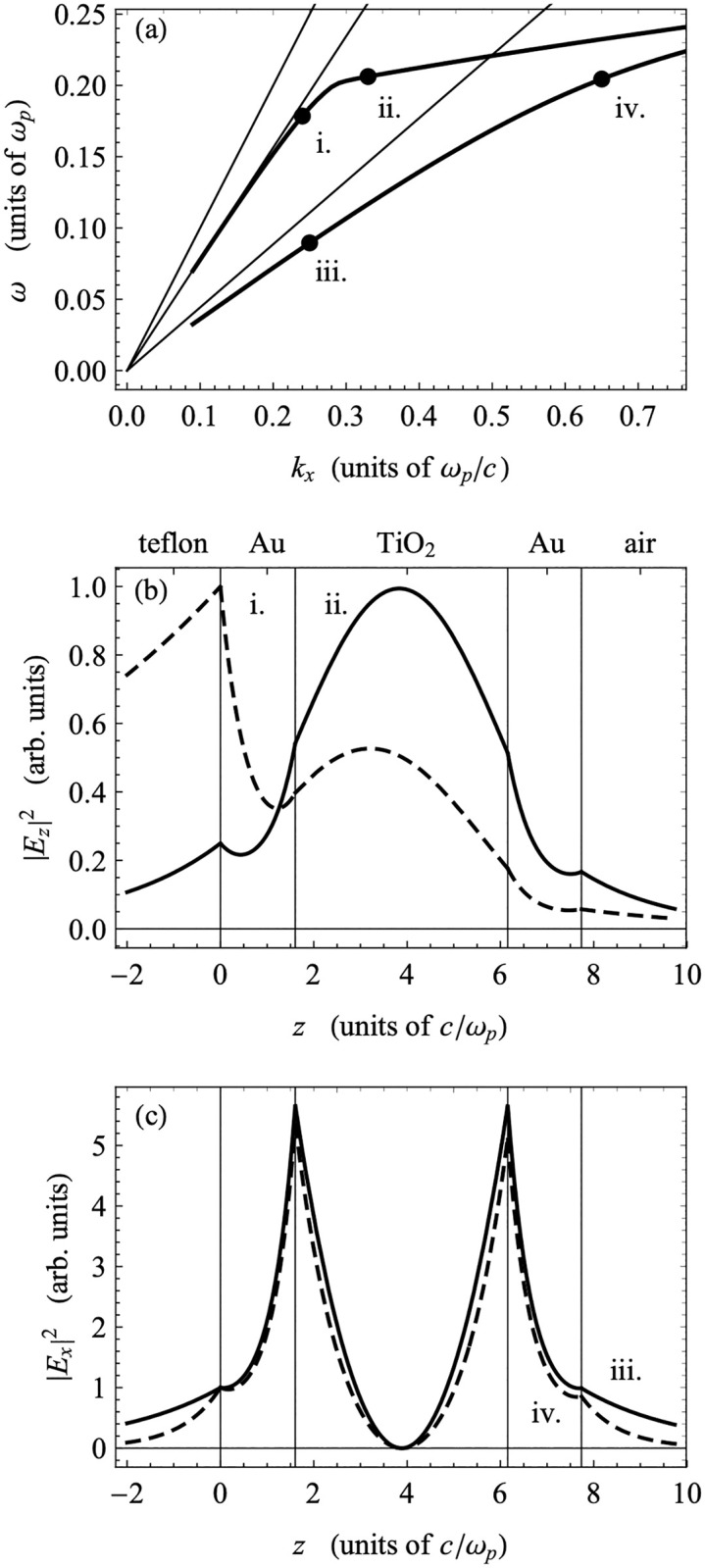
Magnification of lower frequency dispersion region in Fig 6 and electric field amplitudes. a) the lower frequency region of the dispersion displayed in Fig 6; b) electric field amplitude (modulus squared) associated with points i and ii on the dispersion plot; c) electric field amplitude (modulus squared) associated with points iii and iv on the dispersion plot.

Based on the standard approach described above, we calculated the propagation lengths as a function of frequency using the damped dispersion results for the experimentally relevant region of phase space. These are shown in Fig 8, together with the corresponding section of the undamped dispersion. The propagation lengths are largest for the modes closest to the Teflon light line, and they decrease as the frequency of the modes increases. At the same time, as we can see in Fig 7a and 7b, the modes with larger frequencies are less localized in the bulk TiO_2_ layer and more localized to the Teflon-Au interface. This indicates that the propagation length is larger when the field is more localized within the TiO_2_ layer and when the mode is close to the light line. The relatively low propagation lengths of the modes shown are not surprising, given that the higher frequency PPMs are typically associated with a lower propagation length due to the high degree of overlap between the electric field profile and the metal film, similar to the case of Short-Range Plasmon Polaritons (SRSPPs) as described in the prior literature. In addition, these modes display an antisymmetric character about the mid-plane of the TiO_2_ layer—evidenced by a zero in the field amplitude—which would be exact if the dielectric constants of the boundary layers were the same. The higher dielectric constant of Teflon breaks the symmetry, which localizes these modes to the Teflon-Au interface. Due to both this symmetry-breaking and the antiysmmetry of these modes, the confinement of the mode to the TiO_2_ layer is reduced, which can reduce the propagation lengths of these modes. (Much more detail about the mode structure of these devices can be found elsewhere [44, 45]). Therefore, we extend our analysis to two lower plasmonic modes observed in the calculated dispersion plot, despite these modes being outside of the experimentally accessible region for the device reported here. In Fig 9, we have plotted the dispersion relations for the lower frequency modes, including the fully damped dielectric function for the gold along with sample field profiles in the guided wave region and plasmon region. The guided wave modes (i. and ii.) show strong localization in the central TiO_**2**_ layer, due in part to the symmetry of these modes about the mid-plane of the TiO_2_, but the field profiles become more localized to the Teflon-Au interface when they are nearer to the light line. The cross-over between these two behaviors occurs at the cusp between points i. and ii. The plasmon modes (iii. and iv.) that are attached to the TiO_**2**_ light line at small *k*_*x*_ show the typical localization to the TiO_**2**_-Au interfaces.

**Fig 10:**
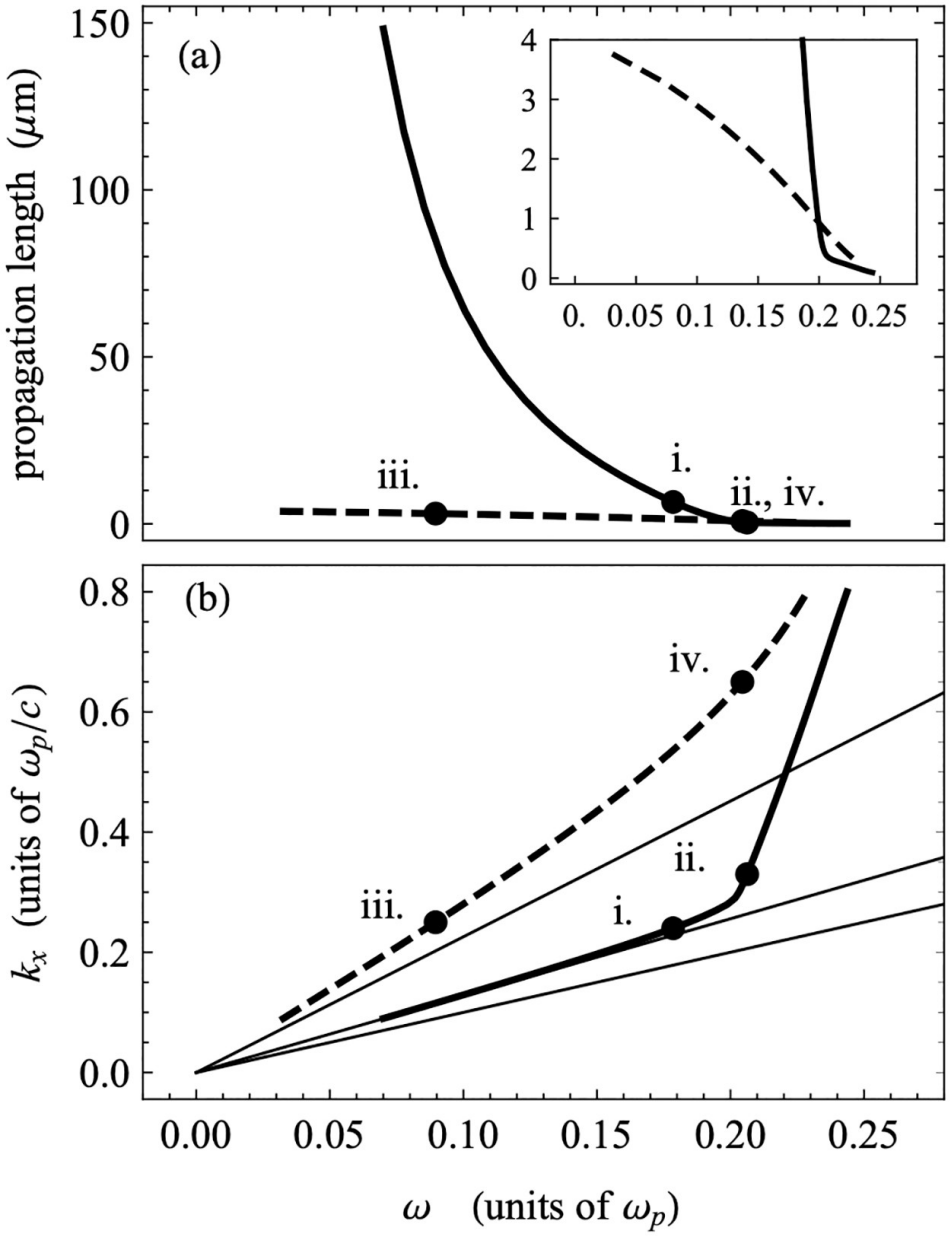
Propagation length and as a function of frequency. a) Propagation length as a function of frequency for the full range of frequencies, as well as focused on the low frequency region (inset); b) A portion of the dispersion displayed in Fig 6, focused on the experimentally relevant region. Several points (i-iv) are shown on both plots for comparison.

In Fig 10, we plot the propagation length as a function of frequency for the two low-frequency branches of the dispersion relation shown in Fig 9a. The propagation lengths in the guided wave region are much larger (≈ 100 ***μm***) for those modes below the cusp, where the dispersion relation is close to the Teflon light line, but decay to below 1 ***μm*** as the frequency of the mode increases past the position of the cusp. In contrast, the modes in the plasmon region have propagation lengths below 1 ***μm*** in nearly the entire range of frequencies, with very low values in the region just below the optical range (which begins approximately around 0.24 ***ω***_***p***_). For reference, propagation lengths for typical SPPs supported by non-symmetric structures like that reported here, determined using a similar method, fall in the range of several microns, opening up the possibility of achieving even higher propagation lengths through the use of a symmetric GW-PPM supporting structure. While the higher propagation length modes in the structure reported here were not accessible in the wavelength/angle ranges available to our experimental setup, it is well-known that changing device parameters such as relative layer thicknesses in the MIM system can significantly shift excitation conditions. Therefore, it likely possible to design a structure in which the higher propagation length mode can be accessed using a typical ATR setup.

## Conclusions

Here, we have shown that the use of a low-refractive index polymer optical coupling layer serves as a viable method of exciting and detecting guided wave plasmon polariton modes (GW-PPMs) using direct reflectance via the Kretschmann ATR configuration. The guided-wave character was confirmed by using ATR measurements to generate a corresponding theoretical model in which the characteristic sinusoidal shape of the electric field amplitude of the excitation can be clearly observed. The calculated propagation lengths for the GW-PPMs accessed using this technique are high, confirming the potential for these modes to improve performance in a variety of plasmonic applications.

## Supporting information

S1 Fig Experimental and theoretically fitted ATR scans, computed without uniform shifts. While the lower- angle scans fit very well, the higher-angle scans display a clear (and relatively uniform across angles) vertical shift.
(TIF)

S2 Fig Experimental and theoretically fitted ATR scans, with vertical shifts added to the higher-angle scans by hand. The parameters used to fit the lower angle scans are also used to plot the higher-angle scans, and by uniformly shifting them down, we can see that the fits match the experimental scans to a high degree of accuracy.
(TIF)

S3 Fig Experimental and theoretically fitted ATR scans, with certain layer thicknesses fixed in the fitting routine. The fits are still very good when the layer thicknesses are set to their values as measured via AFM.
(TIF)

S4 Fig Experimental and theoretically fitted ATR scans, but varying the plasma frequency and damping parameters. The structure of a theoretical scans is highly sensitive to variations in the gold plasma frequency, as shown in the first six panels. There is less sensitivity to the plasma damping, but the width of the resonance feature in the theoretical simulation deviates significantly from the experimental width for values of the plasma damping near the literature value.
(TIF)

S1 File Contains additional analysis of the ATR fitting process. (DOCX)

S2 File Mathematica notebook—ATR fitting. (NB)

S3 File Mathematica notebook—Experimental scan minima. (NB)

S4 File Mathematica notebook—Dispersion calculations. (NB)

S5 File Excel spreadsheet containing experimental ATR data. (XLSX)
