# Supplementary figures and images for: Excitation of “forbidden” guided-wave plasmon polariton modes via direct reflectance using a low refractive index polymer coupling layer
Source: PLoS One. 2022 Oct 26;17(10):e0276522. doi: 10.1371/journal.pone.0276522 (PMC9604954; doi:10.1371/journal.pone.0276522)

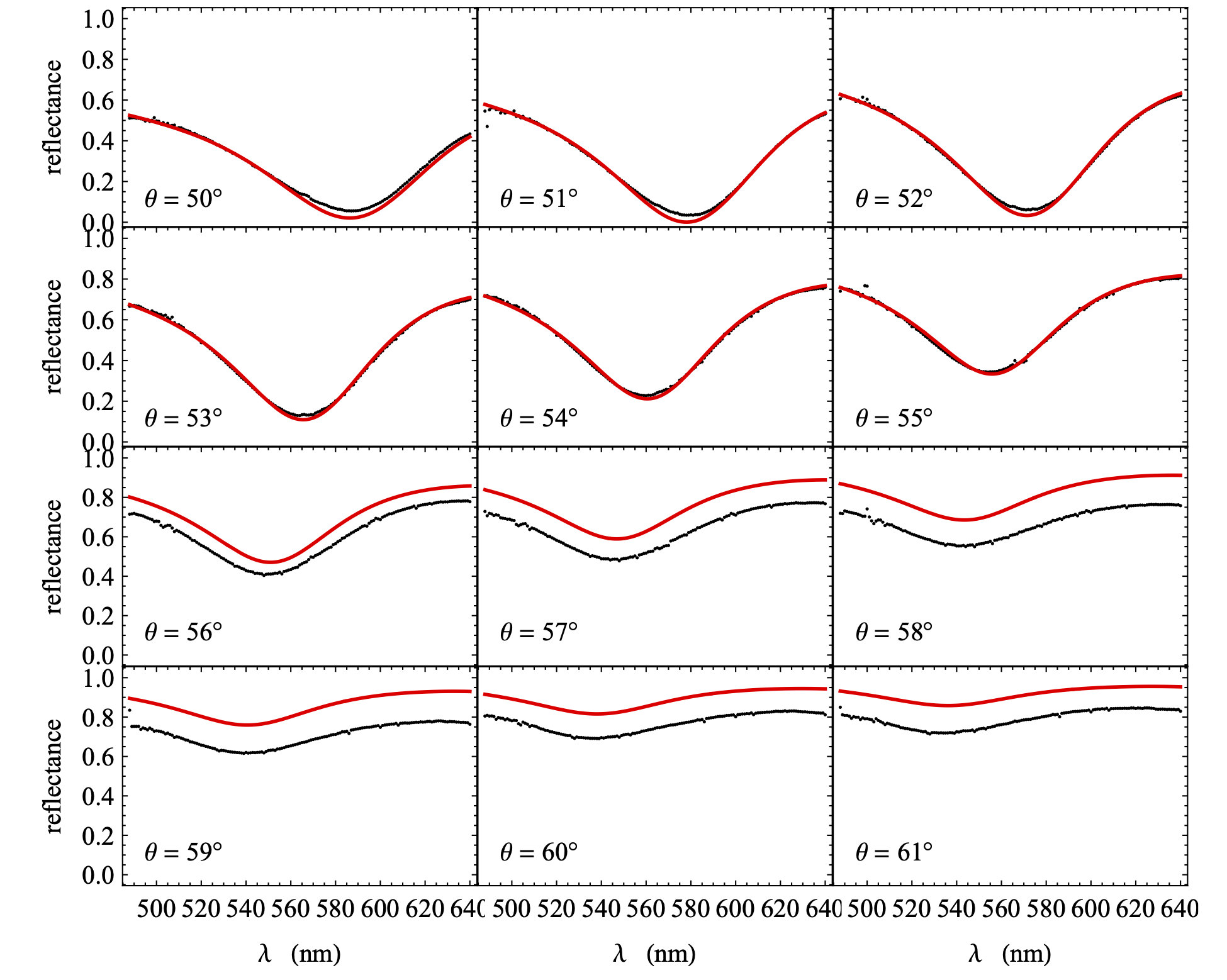

Supplement: S1 Fig — While the lower- angle scans fit very well, the higher-angle scans display a clear (and relatively uniform across angles) vertical shift. (TIF) [file pone.0276522.s001.tif]

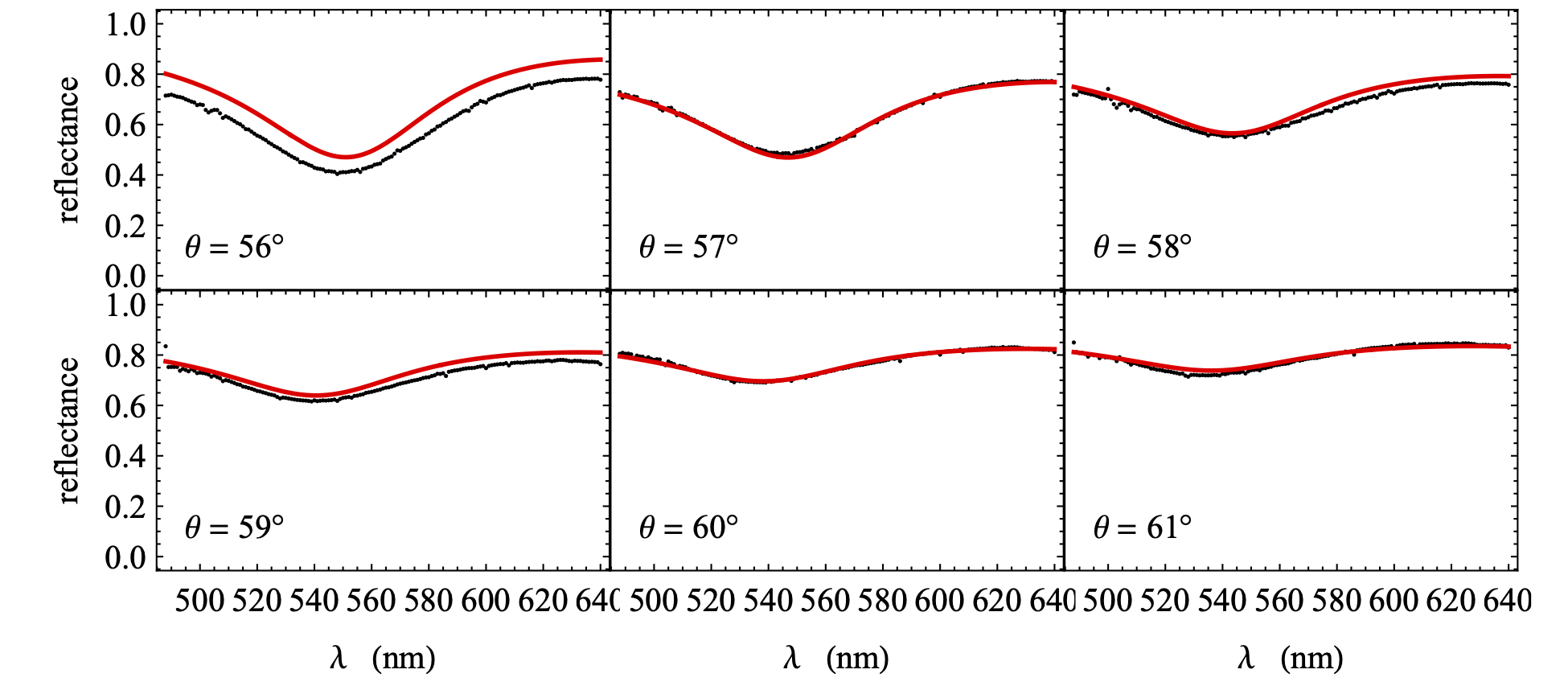

Supplement: S2 Fig — The parameters used to fit the lower angle scans are also used to plot the higher-angle scans, and by uniformly shifting them down, we can see that the fits match the experimental scans to a high degree of accuracy. (TIF) [file pone.0276522.s002.tif]

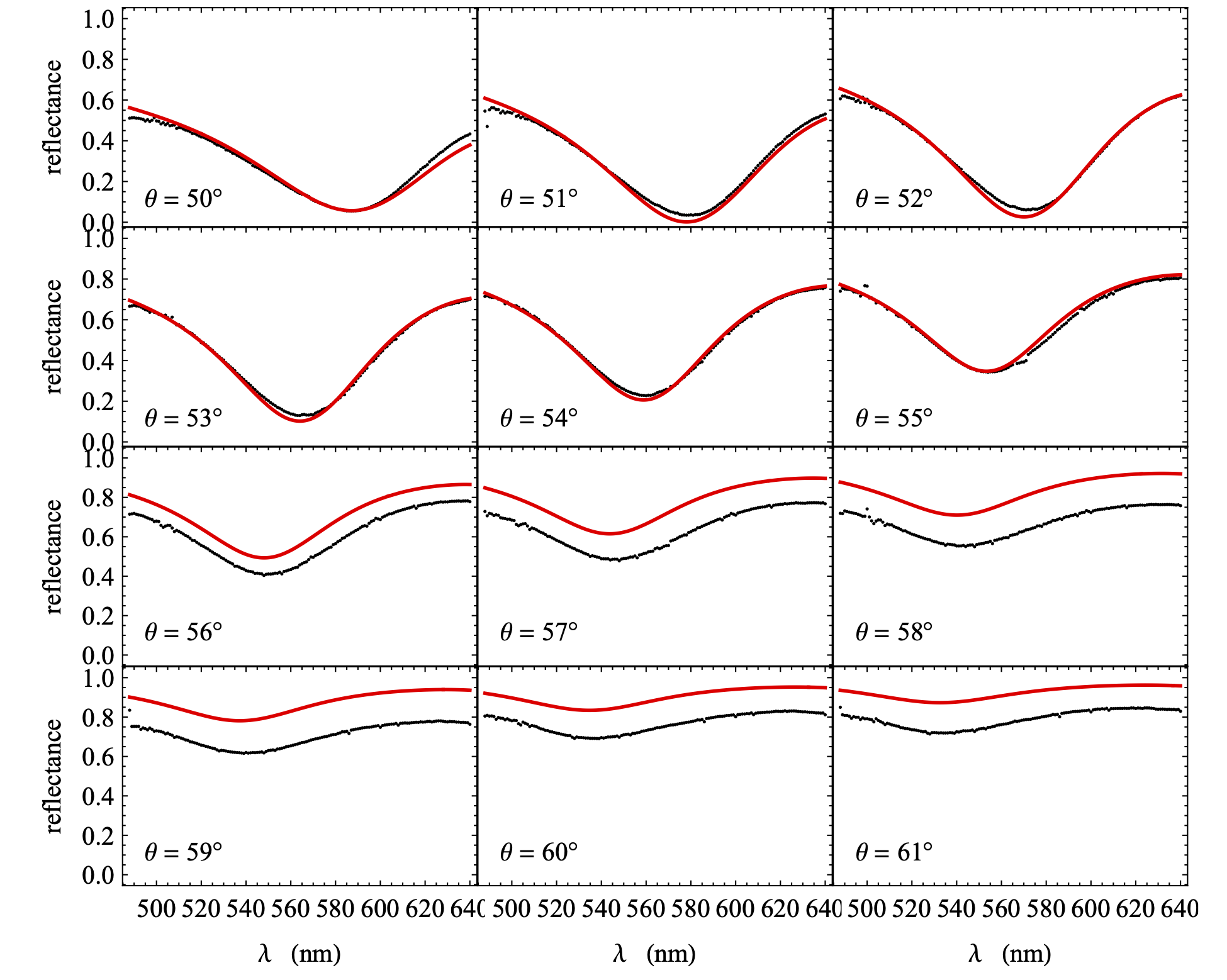

Supplement: S3 Fig — The fits are still very good when the layer thicknesses are set to their values as measured via AFM. (TIF) [file pone.0276522.s003.tif]

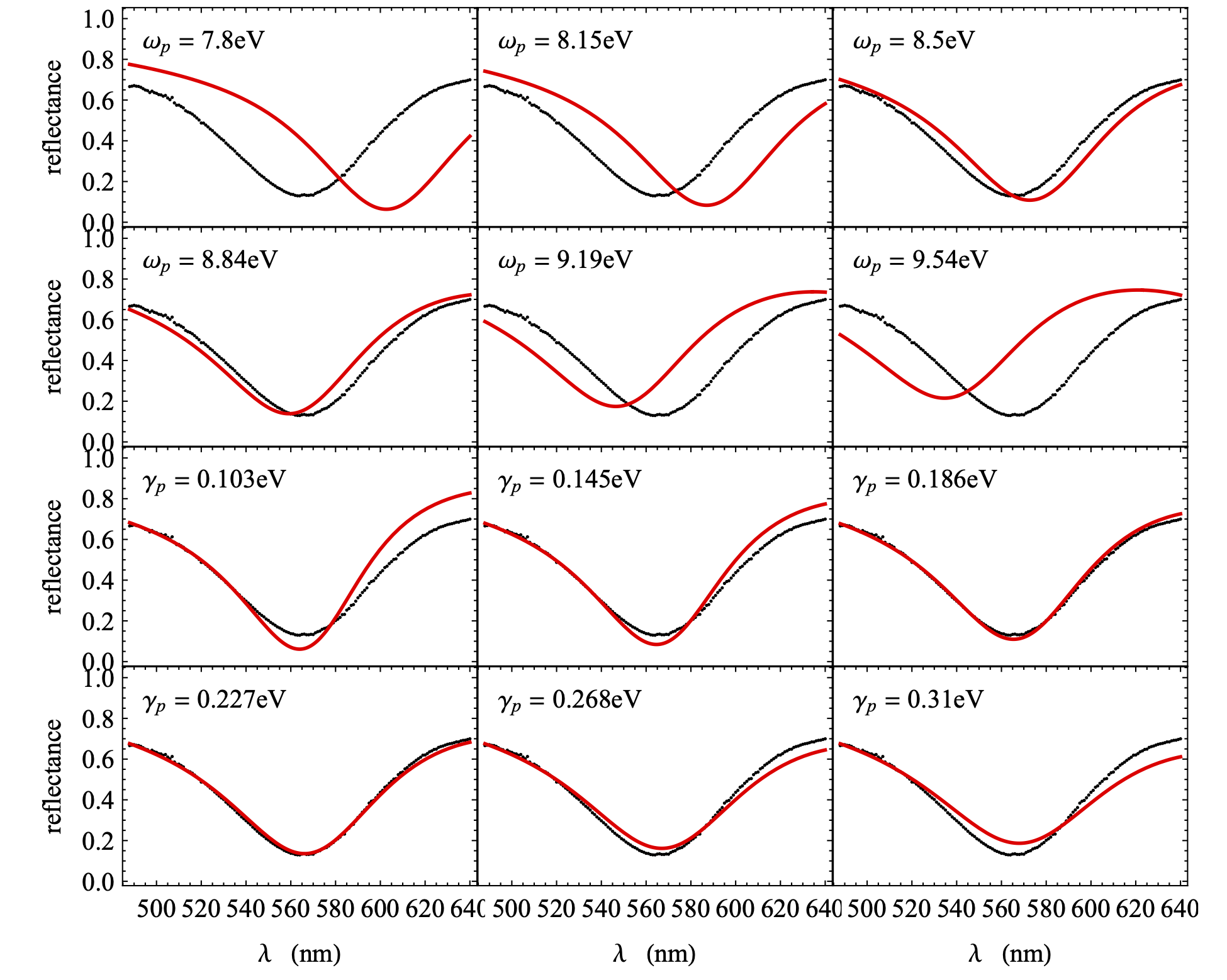

Supplement: S4 Fig — The structure of a theoretical scans is highly sensitive to variations in the gold plasma frequency, as shown in the first six panels. There is less sensitivity to the plasma damping, but the width of the resonance feature in the theoretical simulation deviates significantly from the experimental width for values of the plasma damping near the literature value. (TIF) [file pone.0276522.s004.tif]
