# Supplementary material for: Excitation of “forbidden” guided-wave plasmon polariton modes via direct reflectance using a low refractive index polymer coupling layer
Source: PLoS One. 2022 Oct 26;17(10):e0276522. doi: 10.1371/journal.pone.0276522 (PMC9604954; doi:10.1371/journal.pone.0276522)
Supplement: S1 File — (DOCX) [file pone.0276522.s005.docx]

S1 Appendix – “Excitation of “forbidden” guided-wave plasmon polariton modes via direct reflectance using a low refractive index polymer coupling layer” (Colin D. Marquis, Lindze M. McCarley, Amy L. Pollock, Acamaro S. Cutcher, Max T. Cannella, Tierra L. Smith, Michael B. Larsen, Brandon M. Peden, Brad L. Johnson, Janelle M. Leger)

In this appendix, we describe in more careful detail the procedure use to fit the theoretical AT simulations to the experimental ATR scans in order to extract the physical parameters necessary to compute quantities such as the dispersions relations and propagation lengths for the device. In addition, we justify the final choice of fitting parameters by computing the fits with different sets of fit parameters, thereby extracting good evidence for peculiarities in the experimental data requiring the final choice that we made. This allows us to find good evidence that we are not overfitting the data by including too many (experimentally irrelevant) fitting parameters.

All *Mathematica* code used to both fit the experimental ATR scans and generate the dispersion relations, compute propagation lengths, etc., can be found in the Supplemental Material.

We import the data into *Mathematica* and use a generalization [1] of the built-in function NonlinearModelFit to fit the data. The possible fitting parameters include the parameters in the thicknesses of the layers in the device, the interior angles that the laser light in the prism makes with the prism-Teflon interface, uniform reflectance shifts corresponding to simplified calibration curves for the reflectance, and the model dielectric functions for SF11, TiO_2_, gold, Teflon, and air. The latter include the dielectric constants for air, Teflon and TiO_2_; the parameters in the model dielectric function for SF-11, provided by the manufacturer; and the parameters in the model gold dielectric function, which includes both quantities related to the plasma properties of the free electron gas and quantities related to the two optical transitions in gold [2,3].

In Fig S1, we show the results in which the fitting parameters include the gold plasma frequency and damping, the dielectric constant of the Teflon layer, the angles (limited to 0.3 degrees on either side of the value shown in the figures), and the thicknesses of the TiO_2_ layer and the gold layer between the Teflon and TiO_2_ layers. Importantly, we have simultaneously fit the first *six* scans ($\theta=50^{\circ}$ to $\theta=55^{\circ}$). We observe that the fit to these first six scans is very good, but the fit to the remaining six scans shows a clear upward shift of about 0.1. Indeed, as can be seen in Fig S2, if we uniformly shift the last five scans ($\theta=57^{\circ}$ to $\theta=51^{\circ}$) by 0.12, the fit is again very good. The scan for $\theta=56^{\circ}$ is an outlier in this regard, but as we explain below, we have a plausible explanation for these shifts.

**Fig S1. Experimental and theoretically fitted ATR scans, computed without uniform shifts.** While the lower-angle scans fit very well, the higher-angle scans display a clear (and relatively uniform across angles) vertical shift.

We interpret this observation in the following way. In each scan, the intensity of the incoming light at the interface can be *different* for different angles, for various reasons. One major reason is that due to limitations in the experimental setup, the beam can be clipped at higher angles. This would lead to a shift downward of the measured reflectance that is uniform across all wavelengths, and this is precisely what we see at these higher angles. In addition, this clipping might increase over a small range of angles, which would explain why the scan at $\theta=56^{\circ}$ has a smaller uniform shift than the scans at the higher angles. Ideally, we would experimentally produce a calibration curve at each angle by making an ATR measurement on a blank slide (i.e., with no device so that we are measuring non-attenuated direct reflectance), but this is not feasible given our experimental setup. For this reason, it is reasonable to include uniform shifts as fitting parameters, even though it leads to a large increase in the overall number of fitting parameters. These shifts are *real* in the sense just described, and so they will not contribute to overfitting.

**Fig S2. Experimental and theoretically fitted ATR scans, with vertical shifts added to the higher-angle scans by hand.** The parameters used to fit the lower angle scans are also used to plot the higher-angle scans, and by uniformly shifting them down, we can see that the fits match the experimental scans to a high degree of accuracy.

Having justified the inclusion of the shifts as fitting parameters, we move on to investigate the necessity of the other parameters. First of all, we know that the internal angles can vary by one percent or so, so we use the angles as fitting parameters but constrain them within the numerical routine to vary by no more than $0.3^{\circ}$. The remaining parameters include the layer thicknesses, the Teflon, and the gold plasma parameters. The simulated ATR scans are relatively insensitive to many of the layer thicknesses. For example, in Fig S3, we have plotted the experimental data and simulated scans in which we have fixed the layer thicknesses of both gold layers (both 36 nm) and the Teflon layer. This contrasts with the previous cases shown in which the thickness of the gold layer between the Teflon and TiO2 layers was allowed to vary, and the result was a thickness of 25 nm, which is far from the experimental value of 36 nm. We can see from the plots that the fits are still very good, and for that reason, we can fix many of the layer thicknesses.

**Fig S3. Experimental and theoretically fitted ATR scans, with certain layer thicknesses fixed in the fitting routine.** The fits are still very good when the layer thicknesses are set to their values as measured via AFM.

Finally, we investigate the dependence of the simulated ATR on the plasma parameters in the gold. We chose one set of parameters and then changed by hand the plasma frequency or plasma damping. In the first six panels of Fig S4, we have plotted the simulated ATR reflectance curves for values of the plasma frequency which vary from the fit value within a range of about 10% on either side. It is clear that the ATR is very sensitive to the value of the plasma frequency, and since the literature value was measured for a *bulk* sample of gold [2,3], whereas in this device the gold is a thin film, it is reasonable to expect small deviations from the literature value, and so it is reasonable to use $\omega_{p}$ as a fitting parameter. Finally, we perform a similar analysis for the plasma damping parameter $\gamma_{p}$, whose results are shown in the last six panels in Fig S5. The plasma damping influences the *width* of the resonance dip in the ATR scan, and when $\gamma_{p}$ is relatively close to the literature value (panel 1 in Fig S5), the width of the resonance feature is too small in the simulated scan, although not by a large amount. The ATR scans are not as sensitive to the $\gamma_{p}$ as they are to $\omega_{p}$. In addition, since the gold is deposited as a thin film, $\gamma_{p}$ will deviate from the bulk value (as in [3,4]) due to physical features of the thin film such as surface roughness. So, while the result for $\gamma_{p}$ of the fitting procedure is a value about twice as large as the literature value, the argument above suggests that it is reasonable to both include this quantity as a fitting parameter and expect that the value can significantly deviate from the literature value.

**Fig S4. Experimental and theoretically fitted ATR scans, but varying the plasma frequency and damping parameters.** The structure of a theoretical scans is highly sensitive to variations in the gold plasma frequency, as shown in the first six panels. There is less sensitivity to the plasma damping, but the width of the resonance feature in the theoretical simulation deviates significantly from the experimental width for values of the plasma damping near the literature value.

**References**

^1^ Smit, Sjoerd. MultiNonlinearModelFit, Wolfram Function Repository, <https://resources.wolframcloud.com/FunctionRepository/resources/MultiNonlinearModelFit/>

^2^ Etchegoin PG, Le Ru EC, Meyer M. An analytic model for the optical properties of gold. J Chem Phys. 2006;125:164705. doi: 10.1063/1.2360270.

^3^ Etchegoin PG, Le Ru EC, Meyer M. Erratum: “An analytic model for the optical properties of gold”. J Chem Phys. 2007;127:189901. doi: 10.1063/1.2802403.
